# Exercise and Mental Health: What did We Learn in the Last 20 Years?

**DOI:** 10.3389/fpsyt.2014.00066

**Published:** 2014-06-13

**Authors:** Andrea Camaz Deslandes

**Affiliations:** ^1^Neuroscience of Exercise Lab (LaNEx), Psychiatric Institute, Universidade Federal do Rio de Janeiro, Rio de Janeiro, Brazil

**Keywords:** exercise, depression, neurogenesis, mental health, mental disorders

Although an active lifestyle has always been recognized as the best way to achieve health in the entire history of civilization, over the last two decades, the concept of exercise as medicine (1), or as a preventive method, became increasingly accepted. Some authors consider physical exercise as a “polypill” that, besides many benefits in preventing and treating several diseases, has the advantage of not generating adverse responses and of being a low cost alternative compared to drugs, surgeries, and hospitalizations (2). Researches about the protective effects of physical training and of an active lifestyle in the prevention of metabolic and cardiovascular diseases are not altogether new. However, greater attention is being given to the effects of exercise upon the brain, cognitive function, and behavior (3–5). Since the classic study conducted by Van Praag (6), where the authors showed for the first time that animals submitted to voluntary exercise had an increased neurogenesis, exercise has been investigated as a potential enhancer of cognitive and behavioral functions. Fifteen years later, studies on animal models continue to unravel various neurobiological mechanisms associated with exercise. In another classic study on the neurobiology of exercise, Dishman et al. (3) showed us several ways to explain the prevention and treatment of diseases (including mental diseases) through physical exercise.

However, randomized control trials continue to progress slowly. Studies have shown behavioral, cognitive, and functional improvements in patients who are undergoing physical training, but few have focused on investigating possible neurobiological mechanisms in humans. A better functional capacity and an active lifestyle have been associated with a lower risk of developing neurodegenerative disorders (7), and mood and anxiety disorders (8–11), but the physiological mechanisms are unclear. Works investigating the acute and chronic effects of exercise on cognitive function show promising results in different age groups (children, youth, adults, and seniors) (12–15). However, many questions remain unanswered, such as the right amount of exercise to improve this protective response, the appropriate type, intensity and duration of the training session, and the minimum weekly frequency and duration of benefits after cessation of activity. It is time to think about what we have learned so far and what we still need to understand about exercise and mental health.

## What Do We Know so Far? Humans are Made to Move

In the evolutionary history of human species, several systems need to move to stay healthy and survive (16). Muscles, bones, and even the brain are made of billions of cells that need stimulation for biomolecular signals to occur. Our hearts need to keep beating in order to pump oxygen and energy substrate throughout the entire human body, as much as the skeletal muscles need to be constantly stimulated to synthesize specific myokines that are necessary to maintain health and metabolic activities (17, 18). The extracellular signaling, through neurotransmitters, hormones, growth factors, cytokines, or even mechanical forces, has to constantly occur in order to inhibit the cellular apoptosis and maintain mitochondrial activity and protein synthesis (2). Physical training can promote this stimulus from the brain to the muscle, or vice versa. Although there is a vast literature on the mechanisms of exercise on physical and mental health, one of the main hypotheses is associated with mitochondria, a critical organelle for the survival and the appropriate functioning of cells and, consequently, of every system.

Mitochondrion is a strong candidate to mediate the relationship between exercise and the reduced risk of frailty and mental illness. It is our powerhouse, generating the chemical energy (ATP) necessary for cell survival. On the other hand, it is also responsible for the production of reactive oxidative species (ROS) related to inflammatory processes, aging, and several metabolic and mental diseases (19, 20). According to Radak (21–23), although exercise is associated with acute increases of ROS, chronic alterations related to adaptation to this kind of stimulus contribute to an increased antioxidant enzymes activity. Moreover, exercise promotes mitochondrial biogenesis and makes enzymes more efficient (oxidant and antioxidant), contributing to improved metabolism, function, and cell survival throughout the entire body (24, 25).

Metabolism and protein synthesis maintenance is another important exercise mechanism for promoting health. Inducing the production of trophic factors (such as BDNF, IGF-I, VEGF, GNF) and neurotransmitters (such as dopamine, serotonin, and norepinephrine) contributes to various brain responses, for instance, increased neurogenesis, angiogenesis, synaptogenesis, and inhibition of caspases (3–6, 26–29). Moreover, the increase of neurotropic factors induces presynaptic signals associated with increased release of neurotransmitters in the synaptic cleft and the resulting growth in synaptic transmission and neuroplasticity (30–34). BDNF also contributes to increased long-term potential (LTP) (35–37). During exercise, besides activating motor circuit areas, associative and limbic areas also benefit from the increased synthesis and release of dopamine. Moreover, the activation of opioid and cannabinoid systems contributes to acute anxiolytic response to exercise (30–34, 38).

## What Should We Do? Type, Frequency, Duration, and Intensity of Exercise: Is More Necessarily Better?

Although many neurobiological mechanisms justify the use of exercise in treating mental illness, it is uncertain how we may prescribe this “polypill.” For example, we know that both aerobic and strength training contribute to improve cognitive function, especially the executive function (13–15, 39), and to reduce depressive symptoms (4, 40, 41). It is also known that this improvement may occur through distinct or redundant neurobiological pathways, such as increased neurotrophins. However, there are currently no evidences indicating if certain exercises, e.g., stretching or swimming, would also be favorable. Moreover, it is known that aerobic exercise at moderate intensity (41, 42) contributes to reducing depression and increasing the rate of remission and response among elderly patients. However, it is unclear if these patients would benefit more from making high-intensity interval workouts. Studies indicate that patients with neurodegenerative diseases such as dementia benefit from aerobic exercises. Recently, our laboratory found that elderly people with Alzheimer’s disease have better response to treatment when performing a 30-min walk on the treadmill at a moderate intensity (43). There is only one study submitting patients with AD to a strength training protocol, which found a favorable response in strength and functional capacity. However, the authors did not investigate clinical issues associated with the disease (44). Therefore, it is not possible to indicate this type of exercise for better response to AD treatment.

## Conclusion

Although there are many neurobiological evidences indicating exercise programs to reduce symptoms and increase treatment response of several mental disorders, there is still a long way to go in clinical randomized control trials. Authors in this area need to make an effort to find evidences that may help to determine a more adequate prescription of exercise (type, duration, frequency, and intensity) for each diagnosis of mental illness.

## Conflict of Interest Statement

The author declares that the research was conducted in the absence of any commercial or financial relationships that could be construed as a potential conflict of interest.
